# Salivary microRNAs for oral cancer and oral potentially malignant disorder triage: a scoping review and evidence map

**DOI:** 10.3389/fsurg.2026.1949232

**Published:** 2026-10-02

**Authors:** Jianbang Ding, Han Jiang, Shanyue Lin, Dinghua Li, Ying Zeng

**Affiliations:** 1 Department of Stomatology, Xiangtan Central Hospital, Xiangtan, Hunan, China; 2 Department of Radiology, The Central Hospital of Xiangtan (The Affiliated Hospital of Hunan University), Xiangtan, Hunan, China

**Keywords:** biomarker validation, diagnostic accuracy, evidence mapping, microRNA, oral potentially malignant disorder, oral squamous cell carcinoma, saliva, scoping review

## Abstract

**Background:**

Salivary microRNAs (miRNAs) have been investigated for oral cancer detection, but comparison with healthy volunteers does not establish performance among patients with oral mucosal lesions. This review maps evidence relevant to discrimination between oral squamous cell carcinoma or oral cancer (OSCC/OC) and oral potentially malignant disorders (OPMDs).

**Methods:**

The evidence was organized as a scoping review with study-specific diagnostic descriptions. Clinical comparators, assay methods, thresholds, participant overlap and data provenance were charted. No estimates were pooled. Searches were executed on 8 September 2026 in PubMed (*n* = 487), Web of Science Core Collection (*n* = 362), Scopus (*n* = 318) and the Cochrane Central Register of Controlled Trials (*n* = 34), with 28 additional records identified through citation chasing and reference-list checking, for a total of 1,229 records. After removal of 412 duplicates and 15 records before screening, 802 titles and abstracts were screened; 54 full-text reports were sought, of which 3 could not be retrieved and 51 were assessed. Sixteen sources were included in the evidence map, comprising 13 primary studies and 3 contextual systematic reviews.

**Results:**

Three diagnostic reports contained five assay-level results for cancer vs. OPMD or oral lichen planus. A four-miRNA panel was reported to have 90% sensitivity and 82.7% specificity in 50 OC and 52 OPMD participants. Two individual miRNAs evaluated in the same 30 OSCC and 30 OPMD participants each had reported sensitivity of 70% and specificity of 60%. An additional oral lichen planus comparison had uncertain ROC analysis denominators and was retained without reconstructed counts. Repeated assays within a cohort were not considered independent studies. Small selected samples, data-derived thresholds, inconsistent reporting and limited independent validation restricted interpretation.

**Conclusions:**

Salivary miRNAs have shown study-specific discrimination between cancer and selected OPMD groups. Evidence does not establish a transportable accuracy estimate or clinical benefit for lesion triage. Prospective evaluation in consecutive lesion cohorts is needed before these assays can inform biopsy, referral or surveillance decisions.

## 1 Introduction

Oral squamous cell carcinoma (OSCC) accounts for most oral cancers and remains an important cause of cancer morbidity. Tobacco, alcohol and areca nut exposure contribute to its burden, with substantial geographic variation (1, 2). Oral potentially malignant disorders (OPMDs) comprise clinically distinct conditions, including oral leukoplakia, erythroplakia, oral lichen planus and oral submucous fibrosis (3, 4). Epithelial dysplasia is a histopathological feature that should be recorded separately from the clinical disorder. Transformation risk differs by disorder, dysplasia grade, lesion characteristics and follow-up duration (5, 6).

Patients with persistent or suspicious oral lesions may require biopsy, specialist referral, repeat sampling or surveillance. Histopathological examination remains central to diagnosis, while lesion heterogeneity can complicate sampling. A salivary test would be clinically useful only if it improved decisions within this pathway. Detecting molecular differences between established cancer and healthy volunteers is an earlier evidential step and does not demonstrate that benefit (7, 8).

Salivary miRNAs are candidates for minimally invasive testing. Studies have examined individual miRNAs and multivariable signatures in cancer, OPMDs and related oral conditions (9–14). Existing syntheses describe a broad diagnostic literature, often dominated by comparisons with healthy controls (15–18). OPMD-focused reviews also highlight variation in specimen handling, disorder definitions and the distinction between current diagnosis and future malignant transformation (19, 20).

The relevant triage question is whether an assay distinguishes cancer from the spectrum of lesions encountered in clinical practice. A cancer-associated miRNA may also vary with inflammation, epithelial dysplasia or fibrosis. Results from different comparators, markers and thresholds therefore cannot be treated as measurements of a single interchangeable test. We map the clinical roles and methodological limitations of the available evidence and present diagnostic results by study, with explicit attention to participant overlap and reconstruction uncertainty. We do not estimate an overall accuracy for salivary miRNAs as a class.

## 2 Methods

### 2.1 Review design and reporting

Reporting follows the PRISMA extension for scoping reviews (PRISMA-ScR) (21), with diagnostic reporting informed by PRISMA-DTA and STARD (22, 23). The evidence is synthesized as a scoping review and evidence map with study-specific diagnostic descriptions; no pooled diagnostic meta-analysis is used in the final synthesis. This review was not prospectively registered, and no protocol was deposited in PROSPERO or another registry before the search was conducted. Search, screening and charting procedures are documented in the Supplementary Methods.

### 2.2 Information sources and search

PubMed and Web of Science Core Collection were the information sources described in the original search. Reference lists of relevant reviews were also used to identify reports. The search concepts covered saliva or oral fluid, miRNAs, and oral cancer or OPMDs. In the updated search, the cancer and OPMD concepts were combined with OR within the disease block so that a record did not have to mention both to be retrieved. Supplementary Methods provide the original query and the platform-specific updated search strategies.

The updated search was executed on 8 September 2026 across four databases—PubMed (NCBI), Web of Science Core Collection (v5.38), Scopus and the Cochrane Central Register of Controlled Trials (CENTRAL)—using the platform-specific strategies reported in full in Supplementary Methods. An additional 28 records were identified on 9 September 2026 through backward and forward citation checking of included reports and relevant reviews, and through targeted Google Scholar queries. No language restriction was applied in CENTRAL; PubMed, Web of Science and Scopus searches were limited to English-language records. Deduplication was performed in EndNote 21 (Clarivate), yielding 412 duplicate records removed; 15 additional records were removed before screening. The reconciled PRISMA 2020 flow diagram is presented as Figure 1.

**Figure 1:**
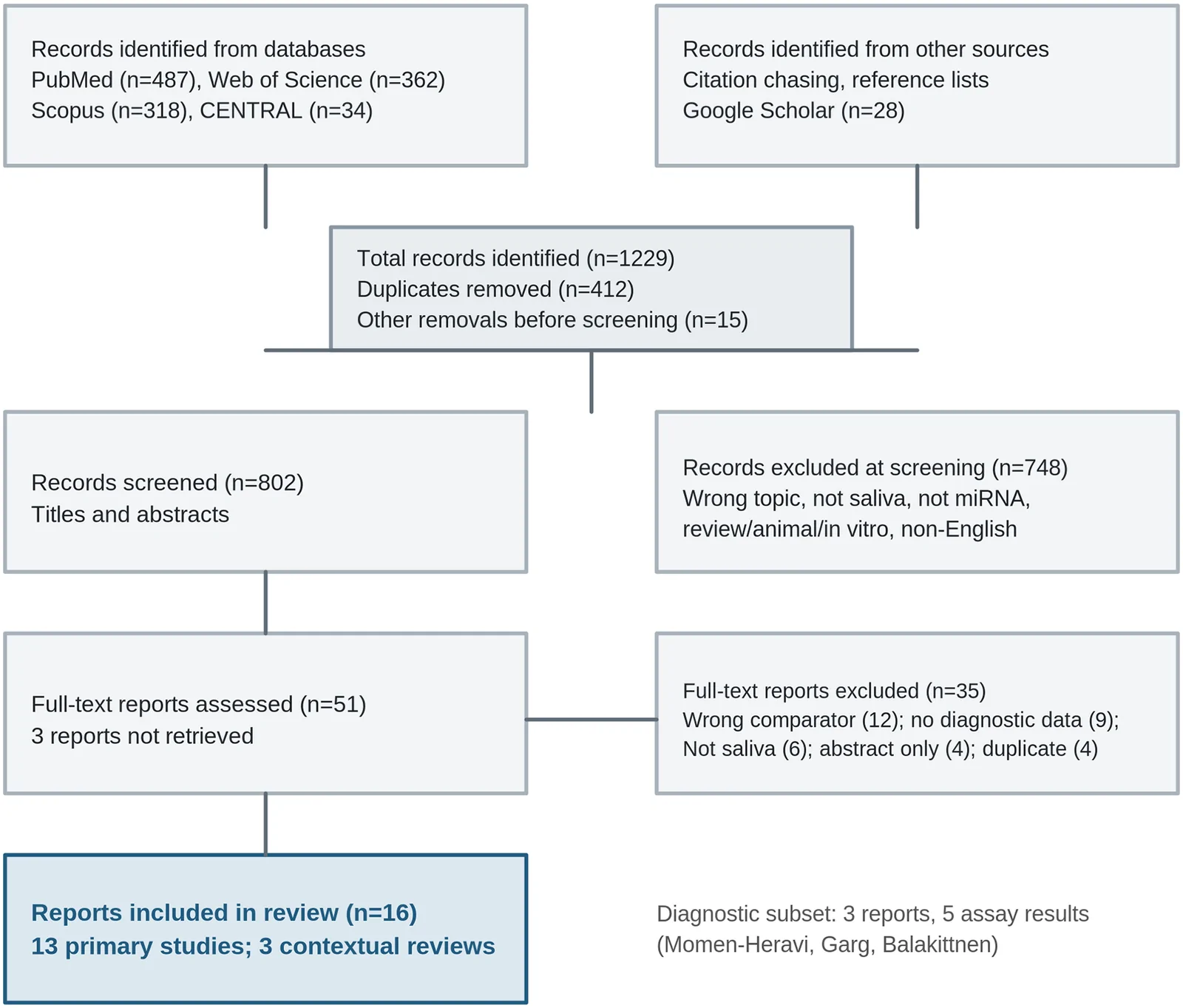
Study identification and selection. Database records identified: PubMed *n* = 487, Web of Science *n* = 362, Scopus *n* = 318, CENTRAL *n* = 34, other sources *n* = 28 (total 1,229). Duplicates removed *n* = 412; pre-screening removals *n* = 15; records screened *n* = 802; excluded at title/abstract *n* = 748; full-text reports sought *n* = 54; not retrieved *n* = 3; assessed *n* = 51; excluded at full text *n* = 35 (wrong comparator 12, no diagnostic data 9, non-salivary matrix 6, abstract only 4, duplicate publication 4). Sixteen sources were included: 13 primary studies and 3 contextual systematic reviews.

### 2.3 Eligibility and selection of sources

The population of interest comprises human participants with OSCC/OC, OPMDs or specified OPMD subtypes. The concept is salivary or closely related oral-fluid miRNA testing, and the context is lesion assessment, diagnosis or longitudinal transformation research. Primary clinical observational studies, including case-control, cross-sectional and cohort designs, are eligible for mapping. A randomized design is not required for a diagnostic evidence map. Purely tissue, serum or plasma studies without a salivary component, non-human studies and reports without miRNA data are outside the primary scope. Reviews are contextual sources and are not counted as primary participant cohorts.

Studies comparing cancer with OPMD or a defined OPMD subtype are charted as direct disease-comparator evidence. Healthy-control comparisons, OPMD subtype comparisons, longitudinal transformation studies and oral-swirl studies are retained in separate roles. Oral lichen planus is not excluded solely because it is a subtype-specific comparator. Diagnostic rates are assigned to a contrast only when that contrast can be identified in the primary report. A numerical ROC result with an unclear comparator or analysis denominator is retained descriptively without an invented two-by-two table.

Two reviewers (JD and HJ) independently screened all titles and abstracts in Rayyan QCRI (https://www.rayyan.ai, accessed September 2026), with reviewer identities masked to each other during screening. Disagreements were resolved by discussion or, when necessary, by adjudication with a third reviewer (SL). Full-text reports were retrieved and assessed independently by the same two reviewers using a standardized eligibility form in Excel (Microsoft 365). Each excluded full-text report was assigned one principal exclusion reason: wrong comparator (healthy-only, *n* = 12), expression-only without diagnostic accuracy data (*n* = 9), non-salivary matrix (*n* = 6), conference abstract without retrievable full text (*n* = 4), or duplicate publication (*n* = 4).

### 2.4 Data charting and source verification

The charting structure records bibliographic identity, country or setting, design, participant groups, sample matrix and fraction, collection and processing details, extraction method, assay platform, miRNA targets, normalization, measurement scale, diagnostic threshold, reference standard, dysplasia information, validation design and clinical utility assessment. Enrolled group sizes are distinguished from the denominators used in a diagnostic analysis. The chart also identifies shared cohorts and whether a number is directly reported, calculated from published aggregate values or not verified. Supplementary Table S1 and the accompanying CSV files expose the fields rather than implying that all variables were completely reported.

The source-level audit transcribed the diagnostic rates and methodological details from the primary reports and cross-checked arithmetic and DOI metadata. A missing or unverified field was not converted into a negative finding. Counts are numbers of participants; sensitivity and specificity are proportions or percentages; AUC, likelihood ratios, diagnostic odds ratios and model probabilities are unitless. Relative qPCR expression and fold changes are assay-specific normalized measurements and are not absolute concentrations. Data charting was performed independently by JD and HJ using a standardized extraction form in Excel (Microsoft 365). Extracted values were cross-checked against the primary reports by SL, and discrepancies were resolved by consensus. Fields that could not be verified from the available full text are explicitly marked NV (not verified). For the Momen-Heravi 2014 miR-27b result, the ROC analysis denominators could not be confirmed from the published report (the reported sensitivity of 85.71% is incompatible with an integer numerator among all 9 enrolled OSCC participants); reported rates are therefore retained without reconstructed counts or confidence intervals.

### 2.5 Appraisal and synthesis

Patient selection, index-test threshold development, reference standard, flow and timing, and applicability were considered using QUADAS-2 domains (24). These source-based methodological observations are presented descriptively. They are not a completed independent QUADAS-2 assessment or a formal GRADE certainty rating. The optional appraisal component of a scoping review does not justify presenting undocumented judgments as a validated risk-of-bias score. Reporting gaps and concerns relevant to clinical transportability are described in the Results and Discussion.

No meta-analysis, pooled sensitivity or specificity, pooled likelihood ratio, pooled diagnostic odds ratio, heterogeneity statistic, funnel plot or pooled post-test probability was retained. Multiple assays measured in the same participants were grouped under one cohort identifier. Identical sensitivity and specificity do not demonstrate identical patient-level classifications. With few heterogeneous cohorts and incomplete data, changing a random-effects variance adjustment cannot remove dependence or make different index tests equivalent. The synthesis therefore describes each study and comparator without a pooled headline estimate (25).

### 2.6 Reconstructed marginal counts and software

For four assay rows from Balakittnen and Garg, marginal two-by-two tables were reconstructed for a transparent arithmetic audit. TP was calculated as floor (sensitivity × number with cancer + 0.5), and TN as floor(specificity × number with the comparator + 0.5). FN and FP were the corresponding residuals. Cancer was treated as the positive reference category. These integers approximate the published aggregate results and are not recovered patient-level classifications. Rates calculated from these integers can differ slightly from the rounded reported rates.

Two-sided 95% Clopper-Pearson intervals were calculated for reconstructed sensitivity and specificity. Study-specific likelihood-ratio and diagnostic-odds-ratio intervals use the conventional log-scale standard errors detailed in Supplementary Methods. These intervals are conditional on the reconstructed counts and do not capture model selection, threshold optimization, participant selection or uncertainty in the original analysis denominators. No continuity correction was required for the reconstructed rows. The Momen-Heravi miR-27b result was not reconstructed because its ROC denominator could not be confirmed. Reconstructed counts and conditional likelihood-ratio and diagnostic-odds-ratio intervals are provided in Supplementary Table S2.

All calculations and figures were generated in R (version 4.4.2) using base and recommended packages. The input tables, executable script, software session information and generated numerical results are supplied in Supplementary Data and Code.

## 3 Results

### 3.1 Evidence roles and selection status

The updated search identified 1,229 records (487 from PubMed, 362 from Web of Science, 318 from Scopus, 34 from CENTRAL and 28 from other sources). After removal of 412 duplicates and 15 records before screening, 802 records were screened by title and abstract. Of these, 748 were excluded and 54 full-text reports were sought; 3 reports could not be retrieved, leaving 51 reports assessed for eligibility. Thirty-five reports were excluded at full text. Sixteen sources were included in the evidence map, comprising 13 primary studies and 3 contextual systematic reviews (Figure 1).

The mapped evidence spans current cancer diagnosis, comparisons with healthy controls, OPMD subtype biology and longitudinal transformation. Table 1 separates these roles. The diagnostic display includes five assay results from three reports: Garg et al., Balakittnen et al. and the OSCC-versus-oral-lichen-planus result reported by Momen-Heravi et al. (11, 13, 14). These three reports form the diagnostic disease-comparator subset and do not represent the entire included evidence base.

**Table 1:** Clinical roles of the mapped salivary-miRNA evidence.

Evidence role	Representative sources	Interpretation
Cancer vs. OPMD or subtype	Garg (13); Balakittnen (14); Momen-Heravi (11)	Direct disease-comparator results are reported separately by assay and cohort. OLP is retained as a defined subtype.
OPMD and cancer expression	Zahran (12); Di Stasio (26)	Relevant clinical populations; expression differences do not automatically supply a reconstructable cancer-versus-OPMD table.
OPMD progression	Hung (27); Tu (28)	Future transformation is distinct from diagnosis of cancer present at sampling.
Healthy-control comparison	Park (9); Liu (10); Romani (36)	Supports discovery and contextual interpretation; does not establish discrimination within a lesion cohort.
Oral-swirl testing	Yap (29)	Retained as adjacent oral-fluid evidence; collection matrix must remain explicit.
Prior evidence synthesis	Rapado-Gonzalez (15); Liu (16); Osan (20)	Context and citation checking only; not independent participant cohorts.

This is a role map of selected evidence sources rather than a participant-level synthesis. One report can inform more than one role. OLP, oral lichen planus; OPMD, oral potentially malignant disorder.

Zahran et al. enrolled 20 OSCC participants, 40 participants with potentially malignant disorders, 20 healthy controls and 20 recurrent aphthous stomatitis controls (12). The report is relevant to the clinical question and is retained in the map. However, the comparator underlying each quoted ROC operating point must be established before its percentages are assigned to an OSCC-versus-OPMD two-by-two table. Di Stasio et al. reported salivary expression across OSCC and dysplasia strata; their dysplasia-detection ROC result is not automatically an OSCC-versus-OPMD accuracy estimate (26).

### 3.2 Study-specific diagnostic findings

Balakittnen et al. evaluated a four-miRNA panel comprising miR-7-5p, miR-10b-5p, miR-215-5p and miR-4707-3p in 50 OC and 52 OPMD participants (14). At a probability threshold of 0.450, the report gives sensitivity of 90%, specificity of 82.7% and AUC of 0.9115. The reconstructed marginal counts were TP 45, FN 5, TN 43 and FP 9. Conditional 95% binomial intervals were 78.2%–96.7% for sensitivity and 69.7%–91.8% for specificity. The report also gives a miR-7-5p-only result with sensitivity of 96%, specificity of 35% and AUC of 0.726 in the same cohort. These are separate index tests, not independent samples.

The Balakittnen panel was selected using LASSO logistic regression with an AICc criterion in the qPCR cohort. The study separated biomarker discovery from qPCR evaluation, but the described panel-fitting procedure does not establish independent external validation of a locked diagnostic model. The reconstructed panel counts imply a positive predictive value of 83.3%, which does not agree with the reported 74.2%. The source values and this arithmetic discrepancy are preserved separately in Supplementary Table S3; neither value is used to claim a transportable clinical predictive value.

Garg et al. evaluated miR-21 and miR-184 in the same 30 OSCC and 30 OPMD participants (13). Both assays were reported to have sensitivity of 70% and specificity of 60% for the OSCC-versus-OPMD contrast. The corresponding thresholds were described as a 2.2-fold increase for miR-21 and a 0.35-fold decrease for miR-184. Each set of marginal rates reconstructs to TP 21, FN 9, TN 18 and FP 12. Conditional 95% intervals were 50.6%–85.3% for sensitivity and 40.6%–77.3% for specificity. The identical marginal rates provide no information about whether the same individuals were misclassified by both assays.

Momen-Heravi et al. reported an OSCC-versus-oral-lichen-planus operating point for miR-27b with sensitivity of 85.71%, specificity of 100%, a cutoff of 14.44 and AUC of 0.9821 (11). The study enrolled nine OSCC and eight oral lichen planus participants. Sensitivity of 85.71% cannot arise from an integer number of true positives among all nine enrolled OSCC participants. We therefore retain the published rates without assigning reconstructed counts or confidence limits from the enrollment totals. Table 2 distinguishes reported values from the conditional calculations, Figure 2 displays the study-specific rates, and Figure 3 shows shared cohort membership.

**Table 2:** Published accuracy and conditional study-specific intervals.

Study and index test	Cancer/comparator n	Reported Se/Sp (%)	Reported threshold	Reconstructed Se then Sp (%) with 95% CI
Balakittnen 2024 Four-miRNA panel	50/52	90/82.7	Probability 0.450; sensitivity fixed at 90%	90.0 (78.2-96.7) 82.7 (69.7-91.8)
Balakittnen 2024 miR-7-5p	50/52	96/35	Numeric cutoff not provided in the reviewed results subsection	96.0 (86.3-99.5) 34.6 (22.0-49.1)
Garg 2023 miR-21	30/30	70/60	2.2-fold increase, as described by the source	70.0 (50.6-85.3) 60.0 (40.6-77.3)
Garg 2023 miR-184	30/30	70/60	0.35-fold decrease, as described by the source	70.0 (50.6-85.3) 60.0 (40.6-77.3)
Momen-Heravi 2014 miR-27b	9/8 enrolled; ROC n unclear	85.71/100	14.44, source-normalized assay scale	Not reconstructed

Se, sensitivity; Sp, specificity. Reported percentages and conditional calculations are deliberately separate. Balakittnen four-miRNA panel AUC 0.9115; single miR-7-5p AUC 0.726; Momen-Heravi miR-27b AUC 0.9821. Garg AUCs were not transcribed from its ROC images. Four reconstructed rows represent two cohorts; the two Garg rows share all participants, as do the two Balakittnen rows. Enrollment totals do not establish the Momen-Heravi ROC denominators. See Supplementary Table S2 for counts and LR/DOR intervals. Sources are references (11, 13, 14).

**Figure 2:**
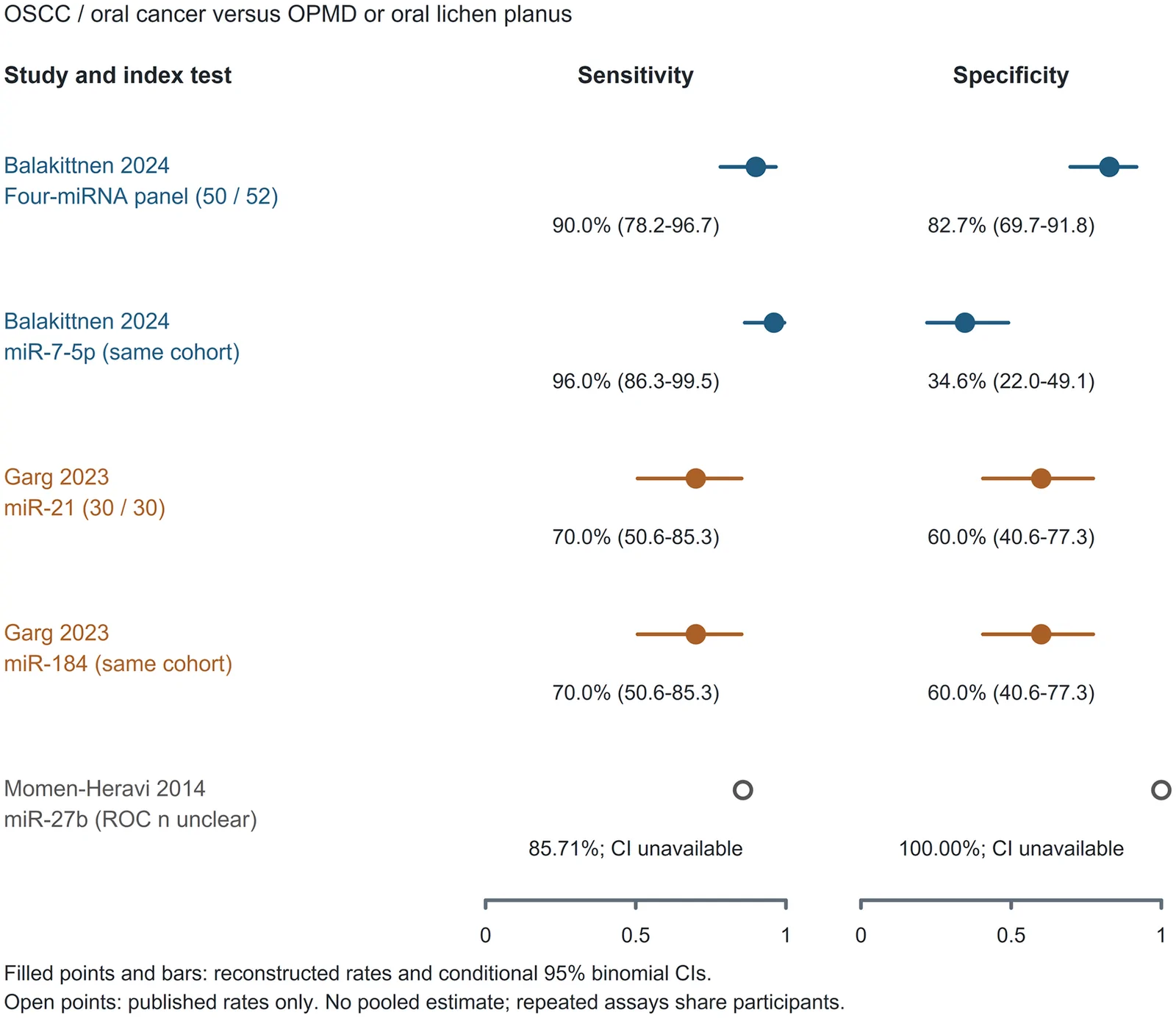
Study-specific salivary-miRNA accuracy for cancer vs. OPMD or oral lichen planus. Each row is an assay result, not an independent study. Numbers in the row labels indicate cancer/comparator group sizes used for reconstruction, except for Momen-Heravi, where the ROC denominators are unclear. Filled points and horizontal bars show rates and two-sided 95% Clopper-Pearson intervals conditional on reconstructed marginal counts. Open points show published Momen-Heravi rates without reconstructed intervals. Balakittnen miR-7-5p specificity is 34.6% after reconstruction from the rounded reported 35%. Repeated rows within each named cohort share participants. No pooled estimate or between-study weighting is shown. Sources are the primary reports and Supplementary Data and Code.

**Figure 3:**
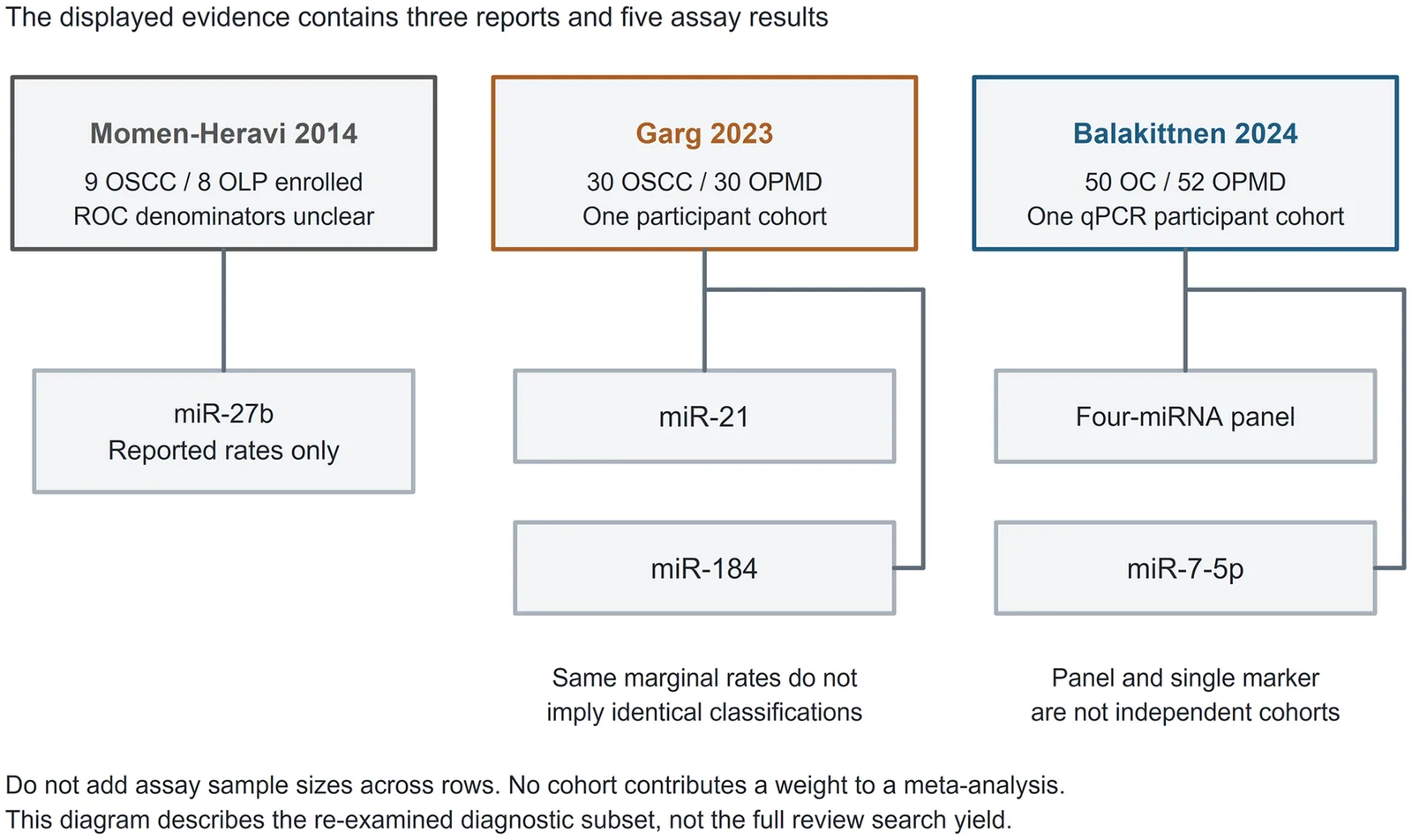
Cohort membership of the displayed diagnostic results. Three diagnostic reports contribute five assay-level results. The two Garg markers share one participant cohort; the Balakittnen panel and single-marker result also share one cohort. Enrollment totals in Momen-Heravi do not establish its ROC analysis denominators. This is a dependency diagram of the diagnostic subset, not a study-selection flow diagram or a complete search yield. No meta-analysis is performed.

### 3.3 OPMD composition and clinical spectrum

The disease comparators differed substantially. Garg included 15 participants with hyperkeratotic or leukoplakic lesions and 15 with oral submucous fibrosis; 26 of 30 OSCC cases were classified as stage IV (13). This spectrum limits applicability to the detection of early cancer among less selected lesions. In Balakittnen, the reported OPMD categories comprised 15 low-grade dysplasia, 12 high-grade dysplasia and 13 lichenoid lesions; these categories account for 40 of 52 participants, leaving 12 not itemized in the source table (14). We do not assign a subtype to the remainder. The Momen-Heravi comparator was oral lichen planus (11). Supplementary Figure S1 preserves the distinctions between these comparator groups.

Expression and transformation studies add information about the disease spectrum without estimating the same diagnostic quantity. Di Stasio reported 10 evaluable OSCC participants, 23 OPMD participants and 10 controls after excluding three inadequate saliva samples (26). Its OPMD group included nondysplastic, low-grade and high-grade lesions. Studies of miR-31 and miR-375 in OPMD address progression-associated biology and should be distinguished from diagnosis of cancer at the time of sampling (27, 28). Oral-swirl signatures likewise require separate interpretation because specimen collection differs from conventional saliva testing (29).

### 3.4 Assay methods and reporting limitations

The diagnostic studies used different normalization and analytical strategies. Garg used qRT-PCR with U6 as the reference and relative expression derived from the 2^(-delta delta Ct) method (13). Momen-Heravi used NanoString profiling with qPCR validation and selected miRNA-191 for normalization (11). Balakittnen combined tissue and salivary discovery data with qPCR evaluation, using the arithmetic mean of miR-191-5p, miR-484 and SNORD96A as reference controls (14). These scales do not support transfer of a numerical cutoff across assays. Table 3 and Supplementary Table S1 report platform, target, normalization, threshold and measurement-scale details, with unverified items identified explicitly.

**Table 3:** Assay and measurement details of the diagnostic subset.

Report	Assay and targets	Normalization and units	Threshold and validation
Momen-Heravi 2014	NanoString nCounter profiling; qPCR validation. Diagnostic result shown for miR-27b.	miRNA-191 selected as endogenous control; normalized expression is assay-specific and unitless.	Data-derived ROC cutoff 14.44 for OSCC vs. OLP. ROC denominators require clarification; no independently locked external test was established by the reviewed report.
Garg 2023	qRT-PCR; salivary miR-21 and miR-184 measured in the same subjects.	U6 reference; 2^(-delta delta Ct) relative expression. Threshold wording is fold change, not a concentration.	ROC-derived operating points. Case-control design; no external evaluation of a locked assay reported in the reviewed methods/results.
Balakittnen 2024	Discovery using TCGA and salivary small-RNA data; miRCURY LNA RT-qPCR. Panel = miR-7-5p, miR-10b-5p, miR-215-5p, miR-4707-3p.	Mean of miR-191-5p, miR-484 and SNORD96A reference Ct values; normalized expression entered into a model. Probability cutoff is unitless.	LASSO model selected by AICc in qPCR cohort; panel probability threshold 0.450. Discovery-to-qPCR evaluation is distinct from external validation of a locked classifier.

Assay nomenclature follows the original reports. A shared numerical cutoff is not transferable between different normalization scales. NR means explicitly not reported after checking the relevant source; NV in the supplementary chart means not yet verified. Sources are references (11, 13, 14).

Methodological concerns include selected participant groups, data-derived operating points, incomplete analysis denominators and uncertain performance of a locked assay in an external lesion cohort. The supplied evidence does not establish improved biopsy selection, referral outcomes, cost-effectiveness or patient benefit. These observations support an evidence-gap map; they are not a formal numerical risk-of-bias score or a completed certainty-of-evidence assessment.

## 4 Discussion

### 4.1 Interpretation for lesion triage

The main contribution of this review is to distinguish several clinical questions that are often combined under salivary-miRNA diagnosis. Studies of cancer vs. healthy controls, cancer vs. OPMD, dysplasia severity and subsequent transformation describe different target populations and outcomes. The disease-comparator studies show that some assays discriminate selected groups, but they do not establish a general performance estimate for all salivary miRNAs or a proven role in clinical triage.

Removing pooled estimates changes the interpretation of the evidence. A precise-looking summary across correlated assay rows could give unwarranted confidence in an average test that does not exist as a single locked laboratory procedure. The alternative is not to select the most favorable marker from each study and combine those results. Study-specific reporting preserves differences in specimen processing, comparator spectrum and threshold development, and makes the substantial uncertainty visible. Small-study interval adjustments or Bayesian priors would not themselves supply independent cohorts, resolve uncertain denominators or establish transportability.

The panel and single-marker findings should also be separated from clinical utility. A marker that classifies established cancers cannot be assumed to reduce missed early cancers or unnecessary biopsies. Even a high sensitivity observed in a selected sample requires validation in consecutive patients with benign, inflammatory, dysplastic and malignant lesions. The present evidence therefore supports research into adjunctive triage, rather than recommending an assay for routine biopsy prioritization or surveillance.

### 4.2 Biological and technical interpretation

Salivary expression may reflect tumor epithelium, non-neoplastic mucosa, immune activity, extracellular vesicles and other oral sources. Differential expression alone does not establish tumor specificity or a causal pathway. The Balakittnen study provides tissue-expression and *in situ* hybridization evidence for selected candidates, which strengthens biological plausibility but does not validate all components of the panel or prove clinical benefit (14). OPMD subtypes and dysplasia grades remain important because inflammation, fibrosis and epithelial transformation can produce different salivary signals (4, 12, 26).

Technical standardization is equally important. Collection conditions, saliva fraction, processing delay, extraction, quality-control criteria and reference genes can alter relative expression. The normalization approaches identified here are not interchangeable. Future work should report MIQE-compatible assay details and evaluate reference-gene stability within the intended specimen and patient population (30–32). Candidate discovery, threshold setting and evaluation of a locked test should be separated. Any new multivariable signature also requires transparent model specification and appropriate validation (33, 34).

### 4.3 Limitations of the review and the evidence

First, the search covered four databases plus citation checking, but the review was not prospectively registered. The original search formulation required both cancer and OPMD concepts to appear together, which may have reduced recall for subtype-specific studies; the updated strategy combined these concepts with OR within the disease block. The classification of Momen-Heravi and the unresolved ROC contrast in Zahran illustrate why the eligibility counts should be interpreted as the yield of the documented search rather than as a definitive census of all possible evidence.

Second, the evidence contains small, selected groups and marked differences in lesion composition. Garg predominantly included advanced cancers, whereas the intended clinical setting includes early malignancy and benign mimics. Subtype-specific comparisons are valuable but cannot be generalized automatically to a mixed lesion population. Third, repeated miRNA measurements within a cohort are correlated. The source reports do not provide the joint classifications needed to estimate the covariance between assay results, and identical marginal rates cannot resolve that problem.

Fourth, the reconstructed tables are conditional approximations. They do not recover the original classifications, and their intervals omit uncertainty introduced by sample exclusions, rounding, model fitting and threshold selection. The Momen-Heravi denominator inconsistency prevents a defensible reconstruction from enrollment totals. The Balakittnen predictive-value discrepancy further illustrates that internally consistent calculations do not necessarily reproduce every reported source metric.

Fifth, the full review-level screening, extraction and independent appraisal procedures require documentary confirmation. Unverified charting fields have not been treated as absent methods, and no formal QUADAS-2 completion or GRADE rating is claimed. Finally, the evidence is largely about discrimination rather than patient-important outcomes. Cross-sectional separation cannot establish prospective transformation prediction, and incident cancers detected shortly after an initial biopsy may represent disease missed at baseline. No pooled post-test probability, clinical threshold or improvement in surgical care is inferred.

### 4.4 Priorities for prospective studies

Future studies should enroll consecutive patients presenting with mucosal lesions, document lesion subtype and dysplasia grade, use a consistent reference standard and retain clinically relevant benign comparators. Investigators should prespecify a reproducible saliva workflow, lock the assay or model before independent evaluation, report exclusions and missingness, and provide complete diagnostic tables with analysis denominators. Evaluation should distinguish discrimination, calibration and clinical utility, including whether using the test improves decisions beyond routine clinical assessment (23, 24, 33, 35). Transformation studies require longitudinal outcomes, time horizons and careful separation of prevalent from incident cancer. Table 4 and Supplementary Figure S2 summarize these priorities. The PRISMA-ScR reporting crosswalk is provided in Supplementary Table S4.

**Table 4:** Evidence limitations and requirements for clinical translation.

Issue	Observed limitation	Required next step
Selection spectrum	Small selected groups; Garg includes 26 of 30 stage IV cancers. Comparator composition differs between studies.	Consecutive pre-biopsy lesion cohorts including benign mimics, OPMD subtypes and early OSCC.
Assay dependence	Repeated tests in the Garg and Balakittnen cohorts do not add independent participants.	Report joint classifications when comparing markers; evaluate a locked assay in independent cohorts.
Data reconstruction	Four rows use rounded marginal counts; Momen-Heravi ROC denominators cannot be inferred from enrollment totals.	Supply analysis-level denominators, exact TP/FP/TN/FN and reasons for exclusions.
Threshold development	Operating points and/or panels were selected from the analyzed data.	Separate development from validation; publish the model and prespecify the operating point.
Reproducibility of the review	The review was not prospectively registered; the original search formulation may have reduced recall, and some source-level charting fields remain unverified.	Future reviews should prospectively register the protocol, preserve complete search and screening records, and prespecify charting and verification procedures.
Patient benefit	No demonstrated improvement in biopsy decisions, referral outcomes or surveillance benefit follows from these accuracy data.	Evaluate incremental value, calibration where relevant, decision utility and patient-important consequences.

These are descriptive concerns, not formal QUADAS-2 scores or GRADE ratings. Recommendations are prospective research priorities and do not imply that the listed work has already been performed.

## 5 Conclusions

Salivary miRNAs have shown diagnostic discrimination in selected cancer and OPMD populations, but the evidence remains heterogeneous and incompletely verified for clinical triage. Study-specific presentation is more defensible than a pooled performance estimate across different and correlated assays. Independent prospective validation is required before a salivary miRNA test can guide biopsy, referral or surveillance. Histopathology remains essential to diagnosis.

## Code availability

The R script reproducing the study-specific calculations and figures, together with its inputs and session information, is included in Supplementary Data and Code.

## Data Availability

The source-level diagnostic inputs, reconstructed aggregate tables, charting fields and audit notes supporting the descriptive results are included in Supplementary Data and Code. These files are extracted or calculated from published reports and are not patient-level data. The updated search strategies, execution dates, PRISMA counts and full-text exclusion categories are reported in the manuscript, Figure 1 and Supplementary Methods.
