# Supplementary material for: Salivary microRNAs for oral cancer and oral potentially malignant disorder triage: a scoping review and evidence map
Source: Front Surg. 2026 Oct 2;13:1949232. doi: 10.3389/fsurg.2026.1949232 (PMC13671942; doi:10.3389/fsurg.2026.1949232)
Supplement: Supplementary file 1 [file Table1.docx]

Supplementary methods and reporting information

Salivary microRNAs for oral cancer and oral potentially malignant disorder triage: a scoping review and evidence map

# 1 Scope and status

This supplement documents the search, selection, charting, source-audit and reproducibility procedures used in the scoping review and evidence map. The diagnostic CSVs and R outputs are computational artifacts supporting the study-level descriptive analyses. The database queries were executed on 8-9 September 2026, and the reconciled PRISMA counts are reported in Figure 1 and in prisma_counts.csv. The procedures reported below correspond to the final evidence map.

# 2 Original search query

The original PubMed query was: (saliva OR salivary OR "oral fluid" OR "oral rinse" OR "oral swirl") AND (microRNA OR miRNA OR miR OR "non-coding RNA") AND ("oral squamous cell carcinoma" OR OSCC OR "oral cancer" OR "head and neck squamous cell carcinoma") AND ("oral potentially malignant disorder" OR OPMD OR leukoplakia OR erythroplakia OR "lichen planus" OR "submucous fibrosis" OR dysplasia OR premalignant). The records available for the original search do not document its execution date, exact export or retrieved counts. The two disease concepts are both mandatory in this original expression, which may reduce recall.

# 3 Updated search strategy

The updated strategy used separate saliva, miRNA and disease concept blocks, with oral cancer and OPMD terms combined by OR inside the disease block. Syntax was adapted to each platform. No randomized-trial filter was applied. Language, date and publication-type restrictions are reported with the search description. Searches covered database inception through the update date. The platform-specific strategies executed on 8 September 2026 are reproduced below.

# PubMed search strategy

("Saliva"[Mesh] OR saliva*[tiab] OR "oral fluid"[tiab] OR "oral fluids"[tiab] OR "oral rinse"[tiab] OR "oral rinses"[tiab] OR "oral swirl"[tiab]) AND ("MicroRNAs"[Mesh] OR microRNA*[tiab] OR miRNA*[tiab] OR "micro RNA"[tiab] OR "miR"[tiab]) AND ("Mouth Neoplasms"[Mesh] OR "oral cancer"[tiab] OR "oral cancers"[tiab] OR "oral carcinoma"[tiab] OR "oral squamous cell carcinoma"[tiab] OR OSCC[tiab] OR "oral potentially malignant"[tiab] OR OPMD*[tiab] OR "oral premalignant"[tiab] OR "oral precancer"[tiab] OR "oral precancerous"[tiab] OR "oral leukoplakia"[tiab] OR "oral erythroplakia"[tiab] OR "oral lichen planus"[tiab] OR "oral submucous fibrosis"[tiab] OR "oral epithelial dysplasia"[tiab])

# Web of Science search strategy

TS=(saliva* OR "oral fluid*" OR "oral rinse*" OR "oral swirl*") AND TS=(microRNA* OR miRNA* OR "micro RNA" OR miR) AND TS=("oral cancer*" OR "oral carcinoma*" OR "oral squamous cell carcinoma*" OR OSCC OR "oral potentially malignant" OR OPMD* OR "oral premalignant" OR "oral precancer*" OR "oral leukoplakia" OR "oral erythroplakia" OR "oral lichen planus" OR "oral submucous fibrosis" OR "oral epithelial dysplasia")

# Scopus search strategy

TITLE-ABS-KEY(saliva* OR "oral fluid*" OR "oral rinse*" OR "oral swirl*") AND TITLE-ABS-KEY(microRNA* OR miRNA* OR "micro RNA" OR miR) AND TITLE-ABS-KEY("oral cancer*" OR "oral carcinoma*" OR "oral squamous cell carcinoma*" OR OSCC OR "oral potentially malignant" OR OPMD* OR "oral premalignant" OR "oral precancer*" OR "oral leukoplakia" OR "oral erythroplakia" OR "oral lichen planus" OR "oral submucous fibrosis" OR "oral epithelial dysplasia")

# CENTRAL Search Manager strategy

#1 (saliva* OR "oral fluid*" OR "oral rinse*" OR "oral swirl*"):ti,ab,kw
#2 (microRNA* OR miRNA* OR "micro RNA" OR miR):ti,ab,kw
#3 ("oral cancer*" OR "oral squamous cell carcinoma*" OR OSCC OR "oral potentially malignant" OR OPMD* OR "oral leukoplakia" OR "oral erythroplakia" OR "oral lichen planus" OR "oral submucous fibrosis" OR "oral epithelial dysplasia"):ti,ab,kw
#4 #1 AND #2 AND #3
CENTRAL is a supplementary source and may have limited coverage of observational biomarker studies. It should not substitute for a broad citation database.

# Citation and Google Scholar checks

Backward and forward citation checking of included reports and relevant reviews, together with targeted Google Scholar searches, was conducted on 9 September 2026. These supplementary searches contributed 28 records to the identification stage.

# 4 Search and selection audit trail

For each information source, the audit trail recorded the database or source, platform, search date, exact strategy or citation-search approach, restrictions and retrieved record count. Duplicate records were reconciled before screening. Each unique record received a title/abstract decision; reports sought for retrieval received a retrieval status, a full-text decision and, when excluded, one principal exclusion reason. Records, reports and studies were treated as distinct units, and multiple reports from one cohort were not counted as independent studies.

# 5 Charting and units

Supplementary Table S1 summarizes the expanded chart. evidence_chart.csv contains all fields. NV means not verified in the current source audit; it must not be interpreted as not reported or not performed. Source-verified absence is described explicitly. Categorical fields have no measurement unit. Group sizes and TP/FN/TN/FP are integer participant counts. Sensitivity and specificity are unitless proportions reported as percentages. AUC, LR+, LR-, DOR and predictive values are unitless. Thresholds retain the source-specific scale, including relative expression, fold change or model probability.

# 6 Reconstructed counts

For eligible rows with stated group sizes, TP = floor(Se × n_cancer + 0.5), FN = n_cancer - TP, TN = floor(Sp × n_comparator + 0.5), and FP = n_comparator - TN. This is nearest-integer rounding with halves upward. The resulting tables approximate aggregate reported rates and do not establish subject-level agreement between assays. For Balakittnen miR-7-5p, rounding 35% specificity with 52 comparators gives TN 18 and specificity 34.615%, which is displayed as 34.6% in reconstructed outputs.

# 7 Interval calculations

Sensitivity = TP/(TP+FN); specificity = TN/(TN+FP). The 95% Clopper-Pearson binomial limits are computed with stats::binom.test. LR+ = Se/(1-Sp); LR- = (1-Se)/Sp; DOR = (TP × TN)/(FP × FN). The standard errors of log(LR+) and log(LR-) are sqrt[1/TP - 1/(TP+FN) + 1/FP - 1/(FP+TN)] and sqrt[1/FN - 1/(TP+FN) + 1/TN - 1/(FP+TN)], respectively. SE[log(DOR)] = sqrt(1/TP + 1/FN + 1/TN + 1/FP). Log-scale intervals are exp[log(estimate) +/- 1.959964 × SE]. No zero-cell correction is applied; the present reconstructed rows contain no zero cells. The script leaves LR/DOR undefined if future input rows have zero cells, rather than silently imposing a correction.

# 8 Interpretation of numerical uncertainty

The binomial and log-scale intervals condition on reconstructed counts. They omit reconstruction, threshold selection, model selection and spectrum uncertainty and are not uncertainty intervals for clinical transportability. The two Garg assays are not averaged. The two Balakittnen assays are not averaged. No meta-analysis, study weights, heterogeneity statistic, funnel-plot inference or pooled clinical probability is computed. Momen-Heravi is retained with published rates only because the ROC denominators cannot be inferred safely from enrollment totals.

# 9 Reproducibility and figure specifications

The executed analysis used R 4.4.2 and base/recommended R packages. Run Rscript reproduce_results.R from the Supplementary Data and Code directory. All calculations are deterministic. The script validates rates, counts, cohort identifiers and the Momen-Heravi denominator incompatibility, and records session information and an input checksum. Figures are 180 mm wide, use Arial text at a minimum of 8.5 points, and are exported as editable-text SVG, PDF, 300-dpi TIFF and PNG. Figure 1 presents the PRISMA 2020 flow based on the documented search and screening records.

# 10 Appraisal and reporting checklist

The review provides source-based methodological observations. A formal independent QUADAS-2 assessment and GRADE rating have not been claimed. The PRISMA-ScR checklist in Supplementary Table S4 maps reporting items to their locations in the final materials.

# 11 Source and policy links

PRISMA-ScR: https://www.prisma-statement.org/scoping
PRISMA 2020 flow: https://www.prisma-statement.org/prisma-2020-flow-diagram
Frontiers author guidelines: https://www.frontiersin.org/guidelines/author-guidelines
Primary diagnostic reports: https://doi.org/10.1038/s41368-023-00273-w ; https://doi.org/10.1007/s12105-023-01600-7 ; https://doi.org/10.1177/0022034514531018
Primary sources were last checked on 11 September 2026. This date is not asserted as the completed search date.
