# Supplementary material for: Salivary microRNAs for oral cancer and oral potentially malignant disorder triage: a scoping review and evidence map
Source: Front Surg. 2026 Oct 2;13:1949232. doi: 10.3389/fsurg.2026.1949232 (PMC13671942; doi:10.3389/fsurg.2026.1949232)
Supplement: Supplementary file 2 [file Table2.docx]

Supplementary tables

Salivary microRNAs for oral cancer and oral potentially malignant disorder triage: a scoping review and evidence map

## Supplementary Table S1. Expanded evidence chart summary

| **Report** | **Design and participants** | **Platform and targets** | **Normalization and units** | **Role and verification** |
| --- | --- | --- | --- | --- |
| Park et al., 2009 | NV OSCC and controls | NV Targets: Early salivary miRNA discovery markers | Normalization: NV Scale: NV | Biological plausibility and secondary evidence. Original map entry retained; only bibliographic metadata verified unless specified below |
| Liu et al., 2012 | NV Oral carcinoma and controls | NV Targets: miR-31 | Normalization: NV Scale: NV | Secondary OSCC diagnostic evidence. Original map entry retained; only bibliographic metadata verified unless specified below |
| Momen-Heravi 2014 | Convenience-sampled clinical case-control biomarker study OSCC; OSCC in remission; OLP; healthy controls | NanoString nCounter miRNA profiling; qPCR validation Targets: Genomewide profiling; miR-27b disease-comparator result; miR-136 for other contrasts | Normalization: miRNA-191 endogenous control selected using NormFinder Scale: Normalized expression, unitless; not absolute concentration | Direct OSCC-versus-OLP result, report-only; no reconstruction Full-text source checked for listed fields; NV fields remain open |
| Zahran 2015 | Clinical comparison of selected diagnostic groups OSCC; dysplastic PMD; nondysplastic PMD; healthy; RAS disease controls | qRT-PCR (Applied Biosystems) Targets: miR-21; miR-145; miR-184 | Normalization: NV Scale: NV | Disease-comparator expression evidence; exact comparator of ROC operating points requires full-text verification Publisher abstract verified; ROC comparator mapping remains unresolved |
| Hung et al., 2016 | NV Oral premalignant lesions | NV Targets: miR-31 and related OPMD markers | Normalization: NV Scale: NV | OPMD risk-stratification evidence. Original map entry retained; only bibliographic metadata verified unless specified below |
| Yap et al., 2019 | NV Oral cancer screening and premalignant context | NV Targets: Multi-miRNA dysregulation score | Normalization: NV Scale: NV | Exploratory adjacent oral-fluid evidence. Original map entry retained; only bibliographic metadata verified unless specified below |
| Di Stasio 2023 | Consecutively recruited clinical pilot cohort with control group Evaluable OSCC; OPMD; healthy controls | NV Targets: miR-21; miR-27b; miR-181b | Normalization: NV Scale: NV | Subtype/degree-of-dysplasia and expression evidence; dysplasia ROC not treated as cancer-versus-OPMD accuracy Primary full text checked for listed fields; remaining laboratory fields NV |
| Garg 2023 | Case-control OSCC; OPMD; healthy controls | qRT-PCR Targets: miR-21; miR-184 | Normalization: U6 reference; 2^(-delta delta Ct) Scale: Relative expression / fold change, unitless | Direct disease comparator; two dependent assays in one cohort Full-text source checked for listed fields; NV fields remain open |
| Balakittnen 2024 | Discovery and qPCR evaluation; LASSO panel fitted in qPCR cohort OC; OPMD; healthy controls | TCGA and salivary small-RNA sequencing discovery; miRCURY LNA RT-qPCR Targets: Panel: miR-7-5p; miR-10b-5p; miR-215-5p; miR-4707-3p. Single miR-7-5p result also charted. | Normalization: Arithmetic mean reference Ct of miR-191-5p, miR-484, SNORD96A Scale: Normalized delta Ct for assays; unitless fitted model probability | Direct disease comparator; panel and single marker share the qPCR cohort Primary full text and tables checked for listed fields; referenced processing protocols remain open |
| Rocchetti et al., 2024 | NV Saliva and plasma miRNA expression | NV Targets: miR-424, miR-138, and related markers | Normalization: NV Scale: NV | Adjacent expression evidence. Original map entry retained; only bibliographic metadata verified unless specified below |
| Koopaie et al., 2024 | Evidence synthesis Oral lichen planus and OSCC evidence | NV Targets: OLP-associated miRNAs | Normalization: NV Scale: NV | Contextual review; not a primary participant cohort Original map entry retained; only bibliographic metadata verified unless specified below |
| Osan et al., 2025 | Evidence synthesis OPMD transition toward oral cancer | NV Targets: OPMD-associated miRNA evidence | Normalization: NV Scale: NV | Contextual review; not a primary participant cohort Original map entry retained; only bibliographic metadata verified unless specified below |
| Wijaya et al., 2026 | Evidence synthesis OSCC detection across biofluids | NV Targets: Comparative miRNA profiles | Normalization: NV Scale: NV | Contextual review; not a primary participant cohort Original map entry retained; only bibliographic metadata verified unless specified below |
| Romani 2021 | NV OSCC and healthy controls | NV Targets: miR-423-5p and discovery miRNAs | Normalization: NV Scale: NV | Healthy-control diagnostic context Original main-text source retained; bibliographic metadata verified |
| Yang 2013 | NV Oral premalignant lesions | NV Targets: OPMD progression-associated miRNAs | Normalization: NV Scale: NV | Longitudinal transformation context Original main-text source retained; bibliographic metadata verified |
| Tu 2022 | NV OPMD and healthy controls | NV Targets: miR-375 | Normalization: NV Scale: NV | Longitudinal transformation context Original main-text source retained; bibliographic metadata verified |

The full 25-field chart is supplied as evidence_chart.csv. NV denotes information that could not be verified from the available source material and must not be interpreted as evidence that a method was absent. The 16-source chart comprises 13 primary studies and 3 contextual systematic reviews.

## Supplementary Table S2. Reconstructed counts and conditional diagnostic measures

| **Study and assay** | **Shared cohort** | **TP / FN / TN / FP** | **LR+ (95% CI)** | **LR- (95% CI)** | **DOR (95% CI)** |
| --- | --- | --- | --- | --- | --- |
| Balakittnen 2024 Four-miRNA panel | BAL2024 | 45 / 5 / 43 / 9 | 5.20 (2.85-9.49) | 0.12 (0.05-0.28) | 43.00 (13.34-138.60) |
| Balakittnen 2024 miR-7-5p | BAL2024 | 48 / 2 / 18 / 34 | 1.47 (1.20-1.80) | 0.12 (0.03-0.47) | 12.71 (2.76-58.41) |
| Garg 2023 miR-21 | GARG2023 | 21 / 9 / 18 / 12 | 1.75 (1.06-2.88) | 0.50 (0.27-0.93) | 3.50 (1.20-10.20) |
| Garg 2023 miR-184 | GARG2023 | 21 / 9 / 18 / 12 | 1.75 (1.06-2.88) | 0.50 (0.27-0.93) | 3.50 (1.20-10.20) |
| Momen-Heravi 2014 miR-27b | MOMEN2014 | Not reconstructed | Not calculated | Not calculated | Not calculated |

All numerical rows are descriptive reconstructions of published aggregate rates. Intervals are conditional log-scale intervals as defined in Supplementary Methods. No pooled row is present. The two Garg rows have identical marginals but unknown patient-level agreement. The Momen-Heravi ROC analysis denominators remain unresolved. These numbers should not be used as clinical device performance claims.

## Supplementary Table S3. Source-level consistency and interpretation audit

| **Item** | **Source issue** | **Verified observation** | **Handling** |
| --- | --- | --- | --- |
| Balakittnen panel membership | An earlier transcription used miR-21-5p. | Primary Results and Table 4 identify miR-215-5p. | The marker is reported as miR-215-5p in the final manuscript, Table 3, figures, code inputs and evidence chart. |
| Balakittnen predictive values | Reported panel PPV 74.2%; Se 90%; Sp 82.7%; groups 50 and 52. | Reconstructed counts 45/5/43/9 imply PPV 83.3% and NPV 89.6%. | Preserve the reported value and the arithmetic recalculation separately. No alternative denominator or adjustment is assumed. |
| Momen-Heravi comparator | Original evidence map characterized the report as healthy-control discovery evidence. | Source also reports OSCC versus OLP, Se 85.71%, Sp 100%, cutoff 14.44, AUC 0.9821. | Restore OLP result to disease-comparator evidence. Do not count it as a new experiment. |
| Momen-Heravi denominator | Enrollment includes 9 OSCC and 8 OLP participants. | No integer TP among 9 gives 85.71%. | Do not infer 6/7 from the percentage or round to 8/9. Retain the reported rates without reconstructed confidence intervals because the ROC denominators are not verified. |
| Garg assay dependence | Two miRNAs evaluated in the same 30 OSCC and 30 OPMD participants. | Each has Se 70% and Sp 60%. | Display both assays but count one cohort. Equal marginals do not prove identical patient classifications. |
| Zahran ROC contrast | The source includes OSCC, two PMD strata, healthy and disease controls. | The abstract does not unambiguously map each ROC operating point to the intended comparator denominator. | Retain the clinically relevant expression evidence. No disease-comparator two-by-two table is constructed because the exact ROC contrast is not verified. |
| Di Stasio bibliographic identity | Original reference points to an unrelated chemistry article. | Correct record is Cancers 15(1):291; DOI 10.3390/cancers15010291. Online 31 December 2022; issue year 2023. | Correct reference and study label. Dysplasia detection is kept separate from OSCC-versus-OPMD accuracy. |
| Original corpus graphics | Earlier corpus-level graphics described frequencies across a larger curated source set. | A record-level denominator for those frequency graphics could not be verified. | Those frequency graphics are not used in the final evidence map or PRISMA counts. |

This table identifies source and extraction uncertainties explicitly. It does not accuse primary authors of misconduct and does not assume an explanation for a discrepancy that cannot be checked.

## Supplementary Table S4. PRISMA-ScR reporting crosswalk

| **Item** | **Reporting domain** | **Location** | **Status** |
| --- | --- | --- | --- |
| 1 | Title | Title | Identifies scoping review |
| 2 | Structured summary | Abstract | Search executed 8-9 September 2026; totals documented in prisma_counts.csv |
| 3 | Rationale | Section 1 | Present |
| 4 | Objectives | Section 1 | Present |
| 5 | Protocol and registration | Section 2.1 | No prospective protocol or registration; stated explicitly in Section 2.1 |
| 6 | Eligibility criteria | Section 2.3 | Eligibility criteria stated and applied to the evidence map |
| 7 | Information sources | Section 2.2; Supplementary Methods | Information sources and search dates reported; four databases plus supplementary citation searching |
| 8 | Search | Supplementary Methods 2-3 | Platform-specific updated search strategies executed on 8 September 2026 and reproduced in Supplementary Methods |
| 9 | Selection of sources | Section 2.3 | Independent JD/HJ screening; SL adjudication when required; full-text exclusion categories reported |
| 10 | Data charting process | Section 2.4 | Independent JD/HJ charting; SL cross-check; consensus resolution; NV reserved for unverified fields |
| 11 | Data items | Table 3; Supplementary Table S1; CSV | Charted fields and measurement conventions reported in Table 3, Supplementary Table S1 and evidence_chart.csv |
| 12 | Critical appraisal if performed | Section 2.5 | Descriptive source audit only; no formal score claimed |
| 13 | Synthesis methods | Sections 2.5-2.6 | Study-level methods and executable code supplied |
| 14 | Selection results | Section 3.1; Figure 1 | Figure 1: 1229 identified, 802 screened, 51 assessed and 16 sources included (13 primary studies and 3 contextual systematic reviews); full-text exclusion reasons specified |
| 15 | Characteristics of sources | Tables 1-3; Supplementary Table S1 | Characteristics of 13 primary studies and 3 contextual systematic reviews are charted in Supplementary Table S1 and evidence_chart.csv; the diagnostic subset is detailed in Tables 1-3 |
| 16 | Critical appraisal within sources | Table 4; Supplementary Table S3 | Descriptive observations; not formal QUADAS-2 |
| 17 | Individual results | Section 3.2; Table 2; Supplementary Table S2 | Reported and reconstructed results distinguished |
| 18 | Synthesis of results | Section 3 | No pooling; study-level descriptive synthesis of the included evidence |
| 19 | Summary of evidence | Section 4.1 | Present |
| 20 | Limitations | Section 4.3 | Review-level and source-level limitations reported in Section 4.3 |
| 21 | Conclusions | Section 5 | Bounded to present evidence |
| 22 | Funding | Funding statement | Funding statement provided |

Item labels are a concise crosswalk to PRISMA-ScR, not a claim of completed compliance. Primary source: Tricco et al., Ann Intern Med. 2018;169:467-473. doi:10.7326/M18-0850.
